# Cross-domain prediction approach of human lower limb voluntary movement intention for exoskeleton robot based on EEG signals

**DOI:** 10.3389/fbioe.2024.1448903

**Published:** 2024-08-23

**Authors:** Runlin Dong, Xiaodong Zhang, Hanzhe Li, Zhufeng Lu, Cunxin Li, Aibin Zhu

**Affiliations:** ^1^ School of Mechanical Engineering, Xi’an Jiaotong University, Xi’an, Shaanxi, China; ^2^ Shaanxi Key Laboratory of Intelligent Robots, Xi’an Jiaotong University, Xi’an, Shaanxi, China

**Keywords:** lower limb voluntary movement intention, readiness potential, convolutional neural network, transfer learning, cross-domain prediction, exoskeleton robot

## Abstract

**Background and Objective:**

Exoskeleton robot control should ideally be based on human voluntary movement intention. The readiness potential (RP) component of the motion-related cortical potential is observed before movement in the electroencephalogram and can be used for intention prediction. However, its single-trial features are weak and highly variable, and existing methods cannot achieve high cross-temporal and cross-subject accuracies in practical online applications. Therefore, this work aimed to combine a deep convolutional neural network (CNN) framework with a transfer learning (TL) strategy to predict the lower limb voluntary movement intention, thereby improving the accuracy while enhancing the model generalization capability; this would also provide sufficient processing time for the response of the exoskeleton robotic system and help realize robot control based on the intention of the human body.

**Methods:**

The signal characteristics of the RP for lower limb movement were analyzed, and a parameter TL strategy based on CNN was proposed to predict the intention of voluntary lower limb movements. We recruited 10 subjects for offline and online experiments. Multivariate empirical-mode decomposition was used to remove the artifacts, and the moment of onset of voluntary movement was labeled using lower limb electromyography signals during network training.

**Results:**

The RP features can be observed from multiple data overlays before the onset of voluntary lower limb movements, and these features have long latency periods. The offline experimental results showed that the average movement intention prediction accuracy was 95.23% ± 1.25% for the right leg and 91.21% ± 1.48% for the left leg, which showed good cross-temporal and cross-subject generalization while greatly reducing the training time. Online movement intention prediction can predict results about 483.9 ± 11.9 ms before movement onset with an average accuracy of 82.75%.

**Conclusion:**

The proposed method has a higher prediction accuracy with a lower training time, has good generalization performance for cross-temporal and cross-subject aspects, and is well-prioritized in terms of the temporal responses; these features are expected to lay the foundation for further investigations on exoskeleton robot control.

## 1 Introduction

Lower limb exoskeleton robots are walking-assist tools that provide movement assistance and functional enhancements for elderly people, lower limb dyskinesia patients, and heavy manual workers to enable them to adapt to hard or high-intensity work (Hussain et al., 2021; Liu and Ouyang, 2023; Plaza et al., 2023). Although lower limb exoskeleton robots have been a popular topic of research in recent years, they are not used in production and the daily lives of people owing to the fact that some of the key technologies are still immature (Hybart and Ferris, 2023). One of these important technologies is human–robot interactive perception, which is the direct communication channel between humans and exoskeleton robots. These robots can better serve the users only by acquiring their active movement intentions.

At present, the movement intentions of exoskeleton robots are primarily detected from human–robot interaction information through various mechanical sensors. However, such interaction signals cannot be acquired until movements occur, and there is additional time involved in processing the acquired signals to elicit responses from the data processing and mechanical systems; thus, there is a considerable time lag in decoding movement intentions based on these signals. In contrast, autonomously generated bioelectrical signals from the human body, such as electroencephalography (EEG), electromyography (EMG), electrooculography (EOG), and electrocardiography (ECG) signals that are generated by chemical signaling between the cells, may exhibit some features before the onset of motion, thereby providing lead time for data processing and mechanical responses of the robot in decoding intentions (Zhang et al., 2021). There is information transmission during intention generation, and the corresponding movements are executed through the nervous system; the brain processes such information and generates movement intentions in the cortical motor areas. At this point, large numbers of postsynaptic membrane potentials overlap in the vicinities of their generation sites according to a spatial sum law, resulting in the observed EEG signal. It is important to note that movements have not yet been executed in these moments. Next, the intention information is transmitted through multiple levels of nerves to the neuromuscular junctions (motor end plates) to control the contraction and relaxation of muscle fibers while completing the movement; this stage generates EMG signals. Obviously, the EEG signals are a direct manifestation of brain activity and have a greater time-response advantage. Nevertheless, EEG signals are relatively weak and susceptible to interference, which places higher demands on the decoding methods used (Mucarquer et al., 2020; Ghosh et al., 2023).

The brain–computer interface (BCI) helps to build a new communication pathway without the participation of peripheral nerves or muscles, thereby allowing the user to send commands directly to a computer or another external device (Li et al., 2018; Dong et al., 2024). This technology decodes human commands based on the time- and/or frequency-domain features of EEG signals. During body movements, electroencephalographic movement-related cortical potentials (MRCPs) are generated in the bilateral supplementary motor areas, bilateral presupplementary motor areas, bilateral cingulate motor areas, and contralateral M1, which contain rich motor information and have strict time- and phase-locked characteristics (Alder et al., 2020). The feature readiness potential (RP) is a slow negative electrophysiological event-related potential (ERP) in the EEG time domain that was first identified in 1964 and is considered to be a part of the MRCP (Shakeel et al., 2015; Benedetto et al., 2022; Vöckel et al., 2023). The properties of the RP that appears before an evoked or a voluntary movement play important roles in movement intention prediction. Many scholars have investigated RP detection. Researchers from the University of Houston investigated the detection of movement intention from the RPs of stroke survivors during upper limb robotic rehabilitation and achieved an ultimate detection accuracy of 67.1 + 14.6% (Bhagat et al., 2016). Jeong et al. (2017) recognized voluntary walking intention based on RPs from healthy people to control a lower limb exoskeleton robot; these experiments were performed under normal and exoskeleton walking, and the average classification accuracy was 80.7% (Jeong et al., 2017). Wang et al. (2020) analyzed the premovement EEG features in the time and frequency domains, and the MRCP features were extracted and decoded for voluntary finger premovements using discriminative canonical pattern matching (DCPM) with an offline average accuracy of 80.96%. Jochumsen and Niazi (2020) detected six different movement tasks from single-trial MRCPs, and the offline classification accuracies associated with movement intention detection in these tasks were in the range of 80%–90%. The common logic in these studies is that the preprocessed signals are first subjected to feature extraction, following which machine-learning methods are applied for pattern recognition. Although there are several studies on optimizing the preprocessing and feature extraction methods, the detection accuracies of movement intention have generally been between 60% and 90% (Garipelli et al., 2013; Jochumsen et al., 2015; Shakeel et al., 2015; Jochumsen et al., 2017). By analyzing these studies, it is easily found that the accuracies of RP-based detection/prediction results for evoked movement intentions are higher than those for voluntary movement intentions. Further, although offline experiments have achieved good results, online methods are not ideal because the accuracies of the pattern recognition results depend greatly on the effectiveness of feature extraction. In the online testing experiments, the insignificant responses from single-trial RPs render feature extraction ineffective, which in turn lower the detection accuracies. Hence, effective extraction and recognition methods for single-trial RP features are needed urgently.

With the advancements in artificial intelligence technologies, deep learning has been shown to have considerable advantages in BCI decoding because it can automatically learn the deep features of EEG signals as well as extract and classify them, thus simplifying the processing greatly (Roy et al., 2019; Hossain et al., 2023). Some of the typical deep-learning models include convolutional neural networks (CNNs), deep brief networks (DBNs), and deep stacked networks (DSNs). EEG signals have spatiotemporal characteristics, because of which CNNs can remain invariant to the scaling and translation of two-dimensional data; this has specific advantages in EEG data processing and classification. Li et al. (2022) proposed a three-dimensional CNN-based model to decode the ERPs dynamically, which was shown to be more robust than other networks. Huang et al. (2021) used the short-time Fourier transform (STFT) technique to convert preprocessed EEG signals to time–frequency images; then, they proposed a multiscale CNN-based EEG signal classification method to recognize hand movement motor imagery (MI) with an average accuracy of 73.9%, which is higher than those of traditional methods involving artificial neural networks (ANNs), support vector machine (SVM), and stacked autoencoders (SAEs) (Huang et al., 2021). Yang et al. (2022) proposed a two-branch CNN that simultaneously learns the temporal and frequency features of EEG data to decode MI-EEG, and the model achieved an average classification accuracy of 81.3%. These studies show that CNNs can better extract the features of multichannel EEG signals than traditional ANNs, thereby improving the classification performances to a certain extent. However, these CNN models are still not able to achieve good accuracies when handling voluntary-movement-intention-related tasks. Model improvements based on the CNN are therefore necessary.

Several scholars have investigated improved CNNs. Zhang et al. (2019) presented a graph-based hierarchical attention model (G-HAM) by combining attention mechanism with CNN to classify left/right fist opening and closing intentions; they obtained an offline evaluation accuracy of 76.36% with better performance over several state-of-the-art and baseline approaches. Zhang et al. (2020) combined a three-dimensional CNN with a long short-term memory (LSTM) network to recognize five actions with better offline accuracy than traditional feature extraction followed by pattern recognition. Li et al. (2020) introduced a recurrent CNN for MI intention recognition by learning decomposed spatiotemporal representations and achieved an offline decoding performance of 92.31% by using 34 channels and a CNN combined with a gated recurrent unit (GRU) network structure. Yue et al. (2021) proposed an EEG2image-based denoised convnets (EID) scheme to enhance the feature representations of intention recognition tasks that achieved high accuracy but required a lot of time. These studies show that complex network results improve the model performances but require significant training and computation times. Although these results are satisfactory for offline analyses, they cannot be used for online real-time movement intention detection. In addition to the large amounts of time consumed to obtain features with more depth, the EEG signals themselves exhibit large cross-temporal and cross-subject variabilities, which is an issue that has been overlooked in some previous studies. In the present study, movement intention detection/prediction was performed with the aim of controlling an exoskeleton robot that is directly worn on the human body and requires high human fitness. Therefore, the problem of cross-temporal and cross-subject variabilities cannot be ignored, and a method that improves the generalization ability of a deep-learning network is needed.

The transfer learning (TL) method enables knowledge transfer from different but related tasks by utilizing existing knowledge learned from accomplished tasks to help with new tasks (Kai et al., 2020; Li and Xu, 2024). The essence here is to find similarities between the original and new tasks to perform discriminative and stationary information transfer across domains, which is a good method of improving network generalization (Samek et al., 2013). The common TL approaches include instance transfer, feature representation transfer, and parameter transfer. Instance transfer approaches reweight some of the source domain data as supplements for the target domain; feature representation TL aims to encode the shared information across subjects/sessions into feature representations; parameter transfer aims is to find shared parameter information to realize knowledge transfer. Parameter transfer methods are some of the commonly used approaches as their structures can be transferred and adapted based on deep-learning networks. Two-dimensional CNN can acquire the deep features of multichannel EEG signals through temporal and spatial convolutions. Using CNN as the pretraining network in combination with TL can solve the above problems associated with practical application and enhance the model generalization ability; this not only improves the network performance but also reduces the pretraining time of the model in the target domain.

This paper presents a novel CNN combined with TL for lower limb voluntary movement intention prediction based on EEG signals. First, we analyzed the characteristics of the EEG RP features. Then, based on the disadvantages of the features and the needs of BCI practical applications, we introduced a parameter TL strategy based on classical CNNs to predict the intentions of voluntary lower limb movements. We recruited 10 subjects for offline and online experiments to validate the proposed model in terms of the cross-temporal and cross-subject performances, and the onset moment of voluntary movement was precisely labeled using lower limb EMG signals. The remainder of this paper is organized as follows. Section 2 describes the methods of the study. Section 3 presents the experiments. Section 4 describes the results of the experiments. Section 5 presents the discussion of the study. Section 6 summarizes the conclusions of the study.

## 2 Materials and methods

### 2.1 EEG RP of lower limb movement

**FIGURE 1:**
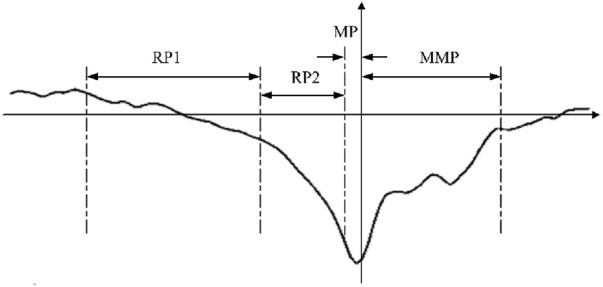
Components of the movement-related cortical potential (MRCP).

The brain generates MRCPs during physical movements, which provides EEG evidence of motor cortical involvement in movement and conscious preparation for the anticipated movements. The MRCP is a low-frequency signal that is readily masked by higher frequency activities and usually has an amplitude of between 5 and 30 μV. As shown in Figure 1, the MRCP can be categorized into three main components as RP, motor potential (MP), and movement-monitoring potential (MMP), which reflect the movement planning/preparation, execution, and control performances, respectively. Among these, the RP is the earliest feature in the MRCP and is essentially an ERP that strictly follows the limb movement in time; its duration varies slightly depending on the form of the movement and individual differences. Based on the waveform, the RP can be categorized into early RP and late RP. The early RP begins about 1.5–2.0 s before motion and is a negative potential with slowly increasing amplitude. The late RP begins about 0.5–1.0 s before movement and manifests as a sharp increase in the negative potential (Shibasaki and Hallett, 2006; Matsuura et al., 2022). The early RP is not easily detected as it occurs early, and intense changes occur in the late RP to MMP phases that can be used to characterize the generation of movement intention.

The MRCP response area mainly corresponds to a large area of the cerebral motor cortex and can be detected over the central electrodes near the midline; however, it is very susceptible to unintentional interference or even erroneous competition among the activated brain regions to the extent that it is difficult to detect in a single instance. Additionally, the timing and amplitudes of the potentials vary with the type of movement, speed of the task, level of uncertainty about the type of movement, preparatory state, and presence of neurological conditions. Some filtering algorithms have been developed for detecting the RP, but only significant RP features can be recognized after superimposed averaging of multiple sets of data; this cannot be used online, so extracting RP features from single-trial data to realize presensing of human movement intention is an important technical difficulty. The ability of the algorithm to extract weak deep features is thus critical.

Furthermore, EEG has some critical disadvantages like low signal-to-noise ratio, non-stationarity, and high individual variability, especially in terms of voluntary EEG signals; these can be affected by noise, environmental disturbances, individual mental states, brain thinking activities, and even EEG cap-wearing errors, making them non-negligible cross-domain problems when using BCI to predict movement intentions. Before predicting any movement intention, a large amount of EEG data is collected from the user to learn the correspondence with the labels to build a subject-dependent network with good performance. However, even the trained network will have degraded performance with the same user the next time it is used. Moreover, individual variabilities can cause the trained network to lack generalization. Both cross-temporal and cross-subject problems can thus cause the performance of the BCI system to be reduced greatly. This necessitates that the algorithm have the ability of decoding weak features as well as good generalization for practical application of the system.

Multilayer CNNs are an effective solution for extracting weak deep features. Compared with traditional machine learning, CNNs use multilayer structures to improve the abstraction performances of the models and achieve good recognition performances, so they have been widely used for image classification in recent years. However, their restricted generalization performances prevent application to BCI systems. Retraining each time with new data to enhance the performance consumes a significant amount of resources and is undesirable. Therefore, we combined the excellent CNN framework with TL to reduce the number of computations while achieving high accuracy and good generalization.

### 2.2 Lower limb movement intention prediction

The problem of movement intention prediction is essentially a pattern recognition problem. The classification performances of deep-learning networks depend on their architectures and complexities. With continuous research, the architectures of some mature CNNs, such as VGGNet, SPPNet, HighwayNet, and R-CNN, have shown advantages for solving different problems with respect to performance (Yujian, 2018).

**FIGURE 2:**
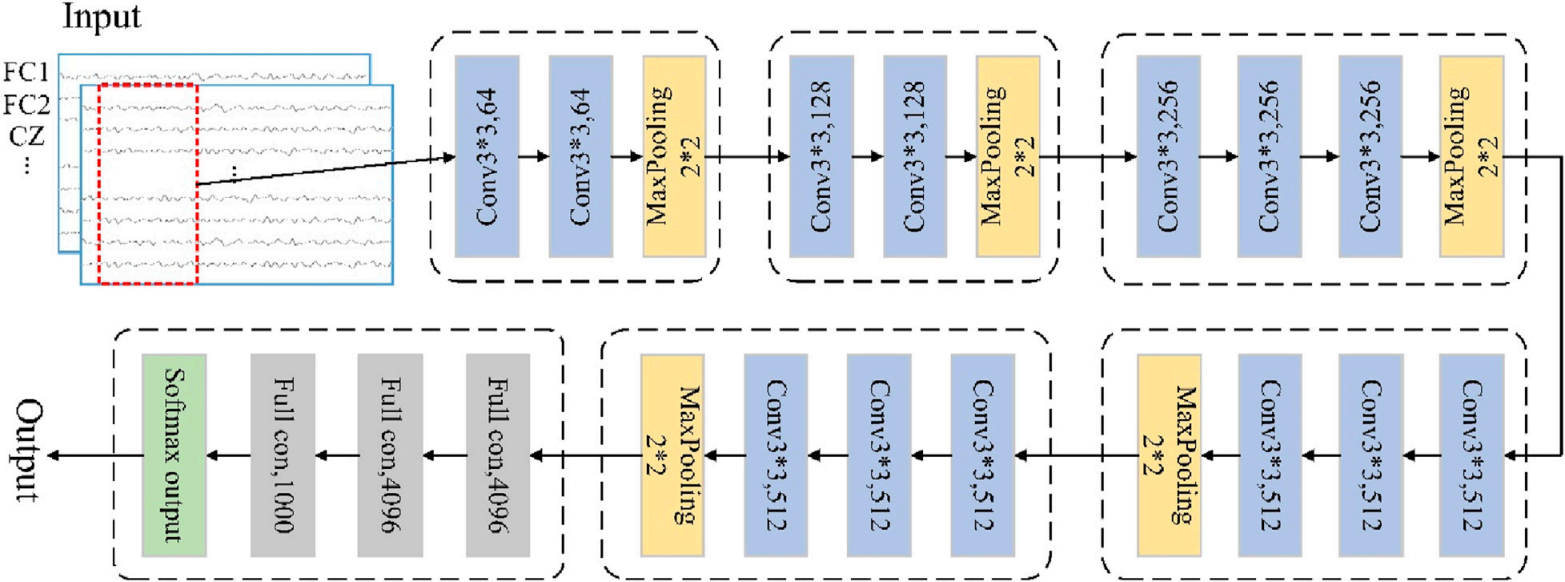
VGG-based convolutional neural network framework.

The VGGNet uses multiple non-linear layers to increase the depth of the network given a small sensory field for the effective region sizes of the input and output layers, thereby enabling it to learn more complex features at lower costs (Quan et al., 2019). This deep-learning approach can be used to find common information between similar classes in non-smooth and non-linear EEG signals, so we use the VGGNet framework for movement intention prediction. The VGG-based CNN model can be regarded as a deepened version of the earlier classical AlexNet model, where the depth of the network is increased by replacing large convolutional kernels with smaller ones to reduce the number of parameters and improve the non-linear mapping ability of the network (Girshick, 2015; Krizhevsky et al., 2017). A typical VGG-based CNN model could have 16 layers, including 13 convolutional and 3 fully connected layers, as shown in Figure 2; here, all layers use 3 × 3 convolution kernels, with rectified linear unit (ReLU) activation, maximal pooling, and identical padding.

**FIGURE 3:**
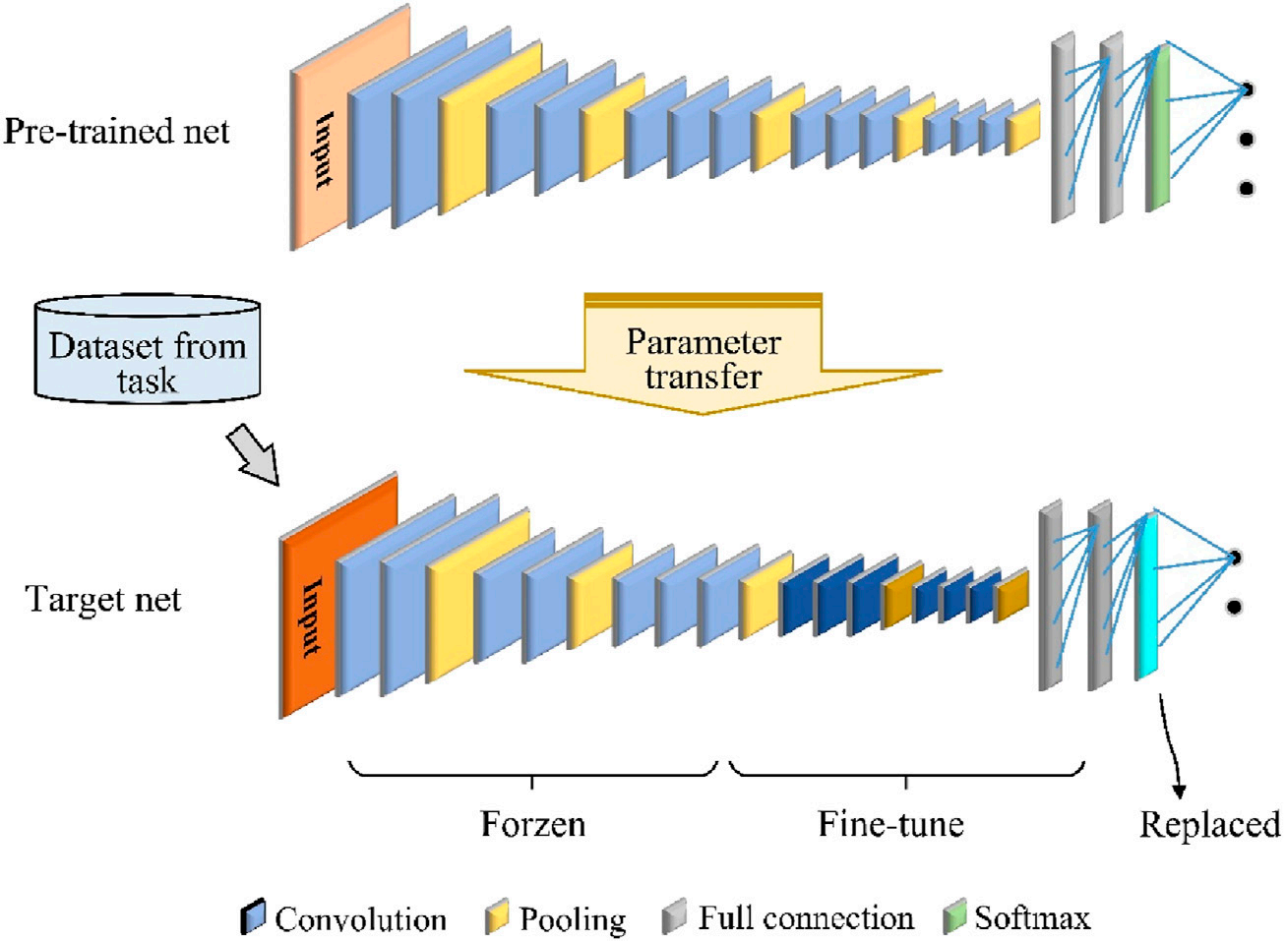
Parameter-transfer-based VGG model.

Using only the VGG network is ineffective because of the problem of insufficient labeling data and time-consuming deep CNN training for cross-temporal or cross-subject movement intention detection based on EEG signals. To address this problem, we used TL to improve the training efficiency of the CNN model with a limited amount of labeled data and limited classifier performance. In the computational process of a deep neural network, the low-level layers are mainly used to learn general-purpose features, and the high-level layers are mainly used to learn specific features related to a particular topic or context (Kai et al., 2020). Therefore, freezing the low-level layers while fine-tuning the high-level layers is a good approach for realizing parameter transfer of a deep-learning model, as shown in Figure 3. The proposed framework consists of a pretrained CNN model and a target CNN model. In this study, we use the above VGGNet framework as the pretrained model.

The target CNN model has the same structure as the pretrained network, except that the original softmax output layer is replaced so that the number of types of output movement tasks is the same. The hyperparameters, parameters, and structure of the pretrained model are transferred to the target model, which is then fine-tuned based on the new data. This improves the model performance without the need to retrain the entire network from scratch. The loss function for fine-tuning the target CNN is softmax cross entropy, defined as Equation 1.

$$H\left(x,p\right)=-\sum_{i}x_{i}\log\left(p_{i}\right)$$ (1)

where *p* is the output probability such that *x* is 1 when the predicted output is the same as the true label and 0 otherwise.

Pretrained weights obtained from training on the original dataset are used as the initial weights instead of random initialization. This helps to utilize the useful features that are already learned by the network while avoiding the limitations of small samples and overfitting. The parameter TL strategy can thus reduce the calibration time required for new tasks and render the CNN suitable for EEG decoding.

## 3 Experiment

### 3.1 Subjects

Ten healthy volunteers (eight males and two females, age: 23–29, mean age: 25 years, referred as S1–S10) without any limb dysfunctions and known cognitive deficits participated in the experiments. None of them had prior experience with the proposed experimental procedures. Before commencement of the experiments, all subjects were introduced to the relevant tasks. This study was approved by the relevant Ethics Committee, and all experiments were conducted in accordance with the principles of the declaration of Helsinki.

### 3.2 Experimental platform

**FIGURE 4:**
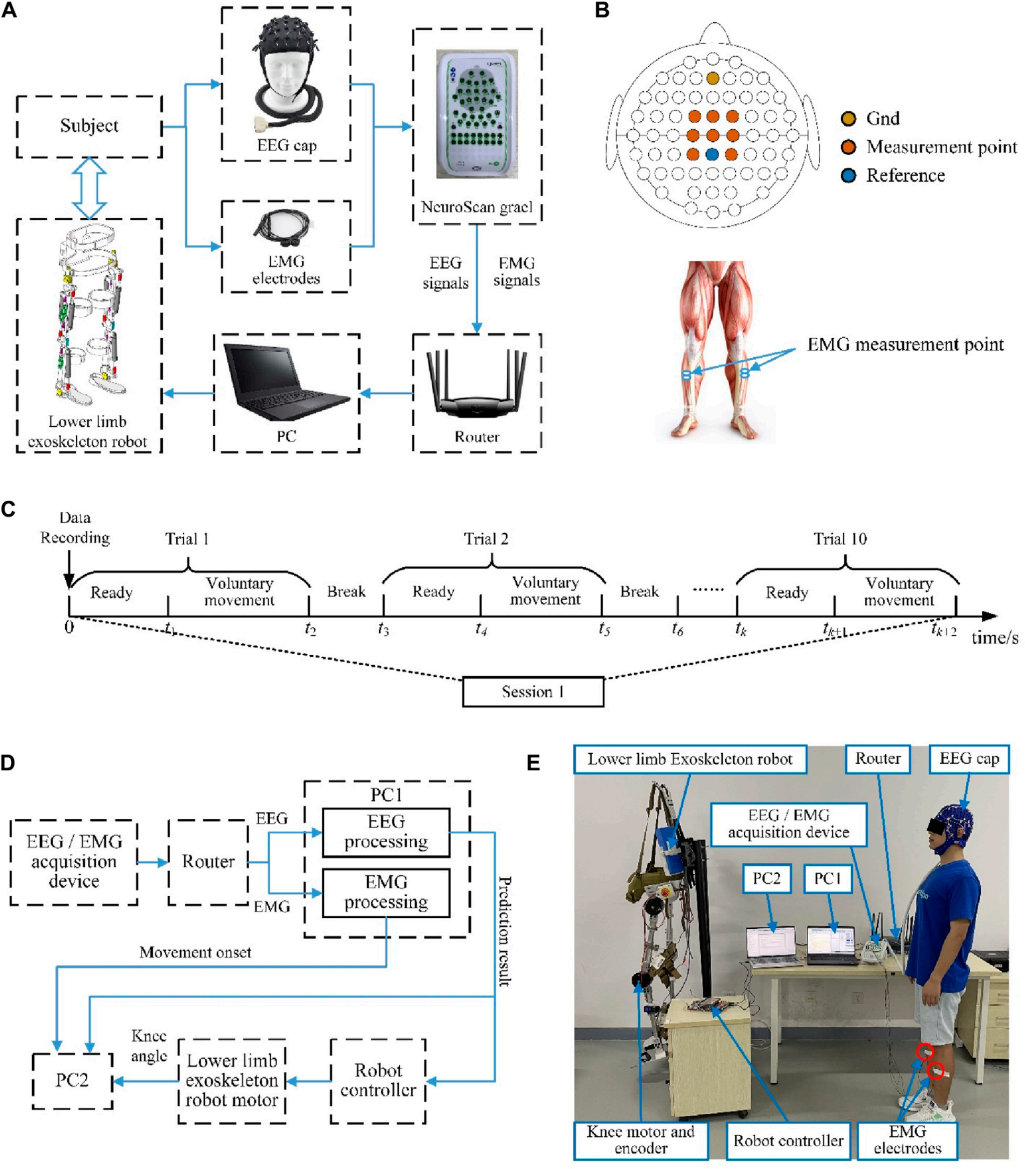
**(A)** Readiness potential (RP) detection and lower limb exoskeleton robot control experimental platform. **(B)** EEG and EMG signal measurement points. **(C)** Overview of the time series of one experimental session. **(D)** Schematic illustration of the online experimental system. **(E)** Photograph depicting the online experimental scene.

The experimental platform was built as shown in Figure 4A and included a synchronized EEG/EMG signal acquisition module, a host computer, and a lower limb exoskeleton robot. A research-grade data recording system (NeuroScan-Grael) was used to capture the brain and muscle activities simultaneously in this study; the system comprises an electrode cap with 32 EEG channels and 8 bipolar channels. A PC with an Intel i5-5600 CPU was used to process the signals. The NeuroScan-Grael system transmits the acquired EEG/EMG data via Wi-Fi to the PC. The algorithmically calculated results are then sent from the PC to the robot controller via Bluetooth, which in turn drives the exoskeleton robot according to the subject’s movement intention.

### 3.3 Data recording and movement perception

In this study, the RPs were detected from the EEG signals, and the onset moment of voluntary movement was determined from the EMG signals. The NeuroScan-Grael system was used for simultaneous acquisition of the EEG and EMG signals at a sampling rate of 1,024 Hz. As shown in Figure 4B, the measurement points of the EEG signals as per the international 10/20 system were Cz, C1, C2, FCz, FC1, FC2, CP1, and CP2, with channels AFz and CPz being used as the references. For each subject, before the experiments, it was necessary to clean the hair, accurately position the EEG cap, and check that the impedance between the electrodes and the scalp was less than 5 kΩ. The lower limb movements were detected by surface EMG signals, and the sampling points were selected from two channels of the tibialis anterior.

### 3.4 Experimental paradigm

We designed both offline and online experiments in this study. The offline experiments were used to train the proposed network and verify the correctness of the algorithm, while the online experiments were used to validate the effectiveness of the proposed method. Each subject had to complete both the offline and online experiments on several separate days, and the experiments were carried out in a quiet and ordinary room without much electronic equipment.

During the offline experiments, the subjects were asked to avoid unnecessary movements in a standing position. All subjects were asked to perform a voluntary movement task that differentiated between left and right leg movements. Each task consisted of five sessions, and the subjects repeated the same voluntary movement tasks for 10 trials during each session. In each trial, the subjects were asked to remain still for at least 5 s and then start walking at will. There were breaks of more than 10 s between each of the voluntary movement trials, and a few minutes of intermission was allowed between every two sessions to minimize the effects of fatigue on the EEG and EMG signals. Figure 4C shows a time series of one session for the offline experiments. To validate the cross-temporal performance of the proposed model, the same experiment was repeated a month later.

The subjects performed the same movement tasks during the online experiment as in the offline experiment but were required to drive the exoskeleton robot based on the decoded movement intentions. Reciprocal exoskeleton robot motions and lower limb movements served as the control targets. Once the control command is detected, it is sent to the exoskeleton robot and a computer for time recording, and the movement onset trigger based on the EMG is also sent to the computer. The prediction system would then not work until the next testing began; this was to ensure that the specific motions of the exoskeleton robot were completed, and all the response times were recorded by the computer. In the online experiments, the prediction system was improved to reflect the prediction performances of lower limb voluntary movement intentions, and another computer (PC2) was added to record all response signals in the system. The computer was mainly connected to the signal processing computer (PC1) through the serial port. The signal processing computer (PC1) sent the prediction results of lower limb voluntary movement intentions based on the EEG and onset of lower limb voluntary movements based on the EMG to the recording computer (PC2). At the same time, PC2 also read the angle values of the absolute encoder in the knee joint of the exoskeleton robot through a USB interface. The framework of the online experimental system is shown in Figure 4D. The subject did not wear the lower limb exoskeleton robot to distinguish the response times between the signals, and the robot performed independent accompanying actions. This experiment reflects the prediction performance of lower limb voluntary movement intention through the comparison of these three response signals. The schematic for the online experiment is shown in Figure 4E.

### 3.5 Experimental data processing

#### 3.5.1 Onset trigger detection of movement based on EMG

For the intention detection/prediction of lower limb voluntary movements, the real movement onset needs to be labeled. Based on previous analyses, it is clear that the EEG and EMG signals are homologous expressions of the same lower limb movements. EMG signals are action potentials generated by the muscle fibers that can directly respond to muscle activity information. Therefore, we used lower limb EMG signals to detect the movement onset trigger points.

**FIGURE 5:**
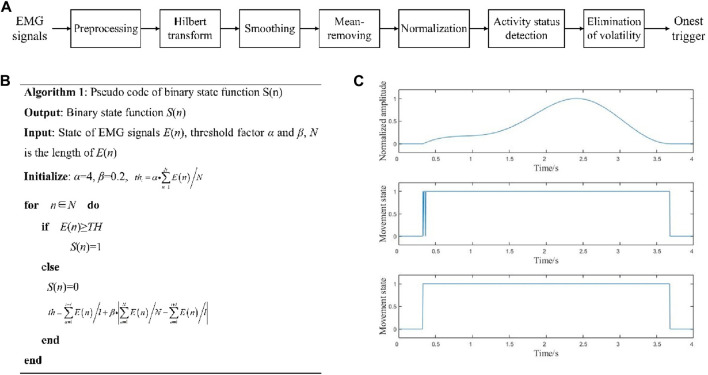
**(A)** Onset trigger detection process; **(B)** pseudocode for movement state determination; **(C)** movement onset trigger detection.

The process of onset trigger detection is shown in Figure 5A. According to the EMG signal characteristics, the data were bandpass filtered in the range of 20–200 Hz with a 4th-order Butterworth digital filter, and the power line frequency was filtered using a notch filter. Thereafter, the EMG signals were Hilbert transformed to obtain the absolute values, as shown in Equation 2.

$$\begin{array}{l} H\left(n\right)=\left|x\left(n\right)*h\left(n\right)\right|\\ h\left(n\right)=\frac{1-{\left(-1\right)}^{n}}{n\pi }=\left\{\begin{array}{l} 0\;\left(n=2,4,6,\cdots \right)\\ \frac{2}{n\pi }\;\left(n=1,3,5,\cdots \right) \end{array}\right. \end{array}$$ (2)

where *x*(*n*) (*n* = 1, 2, … , *N*) is the EMG signal, *h*(*n*) is the discrete Hilbert transform function, and *N* is the length of the signal.

Then, as shown in Equation 3, set the smoothing window *W*(*n*) and calculate the convolution value of the transformed signal *H*(*n*), and *E*(*n*) is obtained after mean-removing and normalization.

$$\begin{array}{l} E_{T}\left(n\right)=H\left(n\right)*W\left(m\right)\\ E_{T}\left(n\right)=E_{T}\left(n\right)-\sum_{n=1}^{N}E_{T}\left(n\right)/N\\ E\left(n\right)=\frac{1-\left(-1\right)}{E_{T}^{\max}\left(n\right)-E_{T}^{\min}\left(n\right)}\times \left(E_{T}\left(n\right)-E_{T}^{\min}\left(n\right)\right)-1 \end{array}$$ (3)

where *E*_*T*_(*n*) is a temporary variable, *W*(*m*) is the smoothing window function, *m* is the length of the smoothing window function (*m* ≈ 0.05*Fs, where *Fs* is the signal frequency), *N* is the length of the signal, and *E*_*T*_^max^(*n*) and *E*_*T*_^min^(*n*) are the maximum and minimum values of *E*_*T*_(*n*), respectively.

Next, the initial threshold *th*_*i*_ was calculated from the signals, and the sliding comparison method was used to judge the state of the signal *E*(*n*). As shown in Equation 4, the binary movement state function *S*(*n*) of the EMG signal was obtained, and *th* was constantly updated according to the data in the sliding window.

$$\begin{array}{l} th_{i}=\alpha \cdot \sum_{n=1}^{N}E\left(n\right)/N\\ S\left(n\right)=\left\{\begin{array}{l} 1\;E\left(n\right)\geq th\\ 0\;E\left(n\right)<th \end{array}\right.\\ th=\frac{\sum_{n=i}^{i+l}E\left(n\right)}{l}+\beta \cdot \left|\frac{\sum_{n=1}^{N}E\left(n\right)}{N}-\frac{\sum_{n=i}^{i+l}E\left(n\right)}{l}\right| \end{array}$$ (4)

where *n*∈[*n*, *n* + *l*], *l* is the length of the sliding window (*l* ≈ 0.05 ∗ *Fs*), and *α* and *β* are both threshold factors (0<*α* ≤ 10, 0<*β* ≤ 1). Here, “1” denotes the EMG in the active state and “0” represents the EMG in the resting state. The pseudocode of this step is shown in Figure 5B.

Finally, the binary state function *S*(*n*) was filtered to eliminate the pseudoactivity detection caused by noise in the active and resting states. This filtering method consists of two steps. The first step involves setting all sequences with a spacing of less than *TA* between two adjacent “1” values to “1” to avoid the occasional resting state due to excessive contraction during muscle activity. The second step involved setting all sequences with spacings less than *TN* between two adjacent “0” values to “0” to eliminate the influences of noise and other spike signals that occur occasionally during muscle rest under normal inactivity. *TA* is the duration of the resting state in normal muscle activity, and *TN* is the duration of the peak pseudoactivity exceeding the threshold in the resting state. Here, *TA* should be as small as possible without affecting the detection of intervals in the EMG signal to avoid advanced detection errors; *TN* should be as large as possible without affecting the duration of normal muscle activity to filter out the noise from the pseudoactivities. Their values were adjusted based on the EMG signals, such that *TA* was about 500 and *TN* was about 1,500 in this work.

After obtaining *S*(*n*), we can detect the movement onset trigger from the EMG signal, which can be marked on the EEG signals. By taking the onset triggers of the lower limb voluntary movements as the data centers, the corresponding data points are defined as the onset times. As shown in Figure 5C, the upper graph is the signal before onset trigger detection, the middle graph is the direct solution to obtain *S*(*n*), and the lower graph is the result after filtering.

#### 3.5.2 Multivariate empirical-mode decomposition (MEMD)-based signal artifact removal method for EEG signals

When processing EEG signals, it is necessary to ensure that the data have high signal-to-noise ratios. The amplitudes of the EEG signals are in the microvolt range, which can be easily contaminated by noise artifacts. Filtering these artifacts is therefore crucial for retaining the valuable information contained in the signals. The MEMD approach based on traditional empirical-mode decomposition (EMD) adopts the Hammersley sequence sampling method to map the target signal onto the hypersphere of the multidimensional space to reduce the problem of mode aliasing between different empirical-mode components; it has the property of being a frequency-independent decomposition, which is suitable for removing the motion artifacts mixed in with the EEG signals. In this study, we established a set of n-dimensional vector sequences as shown in Equation 5.

$${\left\{v\left(t\right)\right\}}_{i=1}^{T}=\left\{v_{1}\left(t\right),v_{2}\left(t\right),\cdots ,v_{n}\left(t\right)\right\}$$ (5)

where *v*(*t*) is the *n-*th element target signal and *T* is the length of the signal sequence.

Then, the Hammersley sequence sampling method was applied to create direction vectors in the *n*-dimensional space on the (*n*-1)-dimensional sphere. Then, as shown in Equation 6, calculate the mapping *P* ^*θk*^ of the input signal *v*(*t*) along each direction vector *X* ^*θk*^.

$$\begin{array}{l} X^{\theta k}=\left[x_{1}^{k},x_{2}^{k},\cdots ,x_{n}^{k}\right]\\ \theta^{k}=\left\{\theta_{1}^{k},\theta_{2}^{k},\cdots ,\theta_{n}^{k}\right\} \end{array}$$ (6)

where *X* ^*θk*^ is the set of direction vectors corresponding to angle *θk* on the (n-1)-dimensional sphere.

We next determine the instantaneous moments *P*_*i*_^*θk*^(*t*) corresponding to the extremum of the mapped signal *P* ^*θk*^(*t*) for all direction vectors, where *i* denotes the position of the extremum point and *i*∈[1, *T*]. Then, the extreme points [*t*_*i*_^*θk*^, *v* (*t*_*i*_^*θk*^)] are interpolated using the multivariate spline interpolation function to obtain *K* multivariate envelopes. For the *K* direction vectors in the sphere space, the *n*-element mean *m*(*t*) is given as Equation 7.

$$m\left(t\right)=\frac{1}{K}\sum_{k=1}^{K}e^{\theta k}\left(t\right)$$ (7)

The intrinsic-mode function *h*(*t*) is extracted as *h*(*t*) = *v*(*t*)-*m*(*t*), and if *h*(*t*) satisfies the multivariate intrinsic-mode function (IMF) judgment criteria, the *v*(*t*)-*h*(*t*) result is treated as the input signal. The above steps are repeated to extract new multivariate IMF components *h*(*t*); otherwise, *h*(*t*) is treated as the input signal. After a series of decompositions, the original *n*-element signal is decomposed into a series of IMF components, and the residual term *r*(*t*) is obtained as a sum. As shown in Equation 8.

$$\begin{array}{l} v\left(t\right)=\sum_{i=1}^{q}h_{i}\left(t\right)+r_{n}\left(t\right)\\ \left\{\begin{array}{l} h_{i}\left(t\right)=\left\{h_{i,1}\left(t\right),h_{i,2}\left(t\right),\cdots ,h_{i,n}\left(t\right)\right\}\\ r_{n}\left(t\right)=\left\{r_{n,1}\left(t\right),r_{n,2}\left(t\right),\cdots ,r_{n,n}\left(t\right)\right\} \end{array}\right. \end{array}$$ (8)

where *q* is the number of IMFs, and *h*_*i*_(*t*) and *r*_*n*_(*t*) correspond to *i* sets of IMF components and *n* residuals of the *n*-element signal, respectively.

**FIGURE 6:**
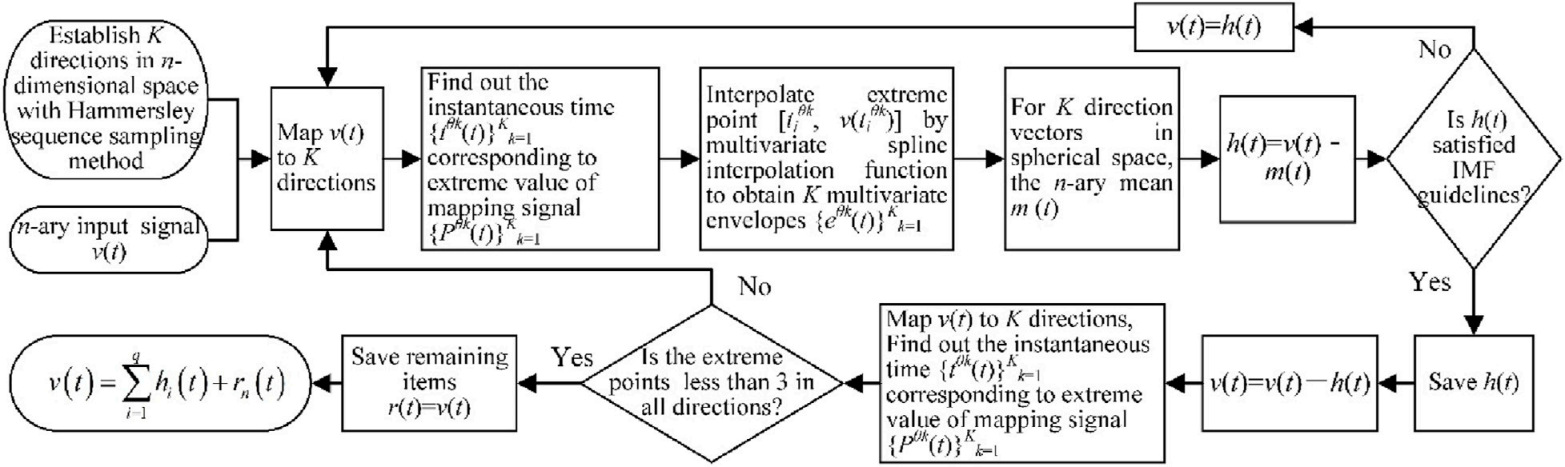
Flowchart of the multivariate empirical-mode decomposition (MEMD) method.

Therefore, the flowchart of the algorithm is shown in Figure 6. The EEG signal in each channel is decomposed into multiple IMF components, and each IMF component signal is Fourier transformed to determine its dominant frequency. According to existing studies, the motion artifacts are mainly in the low frequency range of 0–2 Hz (Song and Wang, 2017); to maximize the retention of the signal itself, this study discards IMF components whose principal frequencies are lower than 0.5 Hz, and the remaining components and residual terms are reconstructed to remove the motion artifacts in the EEG signals.

#### 3.5.3 Data segmentation, training, and testing

After detecting the movement onset triggers based on the EMG signals, the EEG data before and after the onset moment were extracted and aligned for further analyses. The classifiers were trained using the EEG data in the range of −1.0 s to 1.0 s of the actions, which was labeled as the movement intention stage; data in the range of −3.0 s to −1.0 s was labeled as the resting stage. This time window was chosen based on neurological background suggesting that the significant MRCP (late RP to early MMP) originates 0.5–1.0 s before the movement starts and can extend until 1.0 s after the event. This window was only used as the active intention state for offline validation. During the online experiment, the prediction window was set from −1.0 s to the movement onset time; if any intention was predicted after movement onset, it was marked as a false positive result.

The initial 70% of the collected data were used to train the network, while the remaining 30% were used for testing. The input size of the network is set to number of channels × number of datapoints × 1, and the output is categorized under two classes. In all cases, bandpass filtering and baseline calibration were used for fair CNN input-to-output comparisons. In the cross-temporal test, the newly collected data were used as inputs to the trained network based on past data. In the cross-subject test, each subject was tested using a network trained on data from the other subjects. All data training and testing procedures were performed using MATLAB R2019a.

#### 3.5.4 Methods of comparison

To verify the superior performance of the proposed method, the results obtained with SVM and VGGNet were compared. SVM has shown many advantages in solving small-sample, non-linear, and high-dimensional pattern recognition problems which are the most frequently used machine-learning methods in EEG decoding. The core idea of the SVM method is to find a maximally spaced hyperplane that separates the sample points of different classes while maximizing the spacing between the two classes. Specifically, SVM ensures that the data are linearly separable in a high-dimensional feature space by mapping the samples to that space. A kernel function is introduced to shift the computational complexity from the high-dimensional feature space to the original input space to find the hyperplane. The commonly used kernel functions are linear, polynomial, and Gaussian kernels. In this study, we used the SVM classifier with the radial basis function (RBF) kernel as the reference to classify the same task. Moreover, the VGGNet used for comparison has the same structure and parameters as the proposed model, with the only difference being the use of TL.

## 4 Result

### 4.1 RP detection results

**FIGURE 7:**
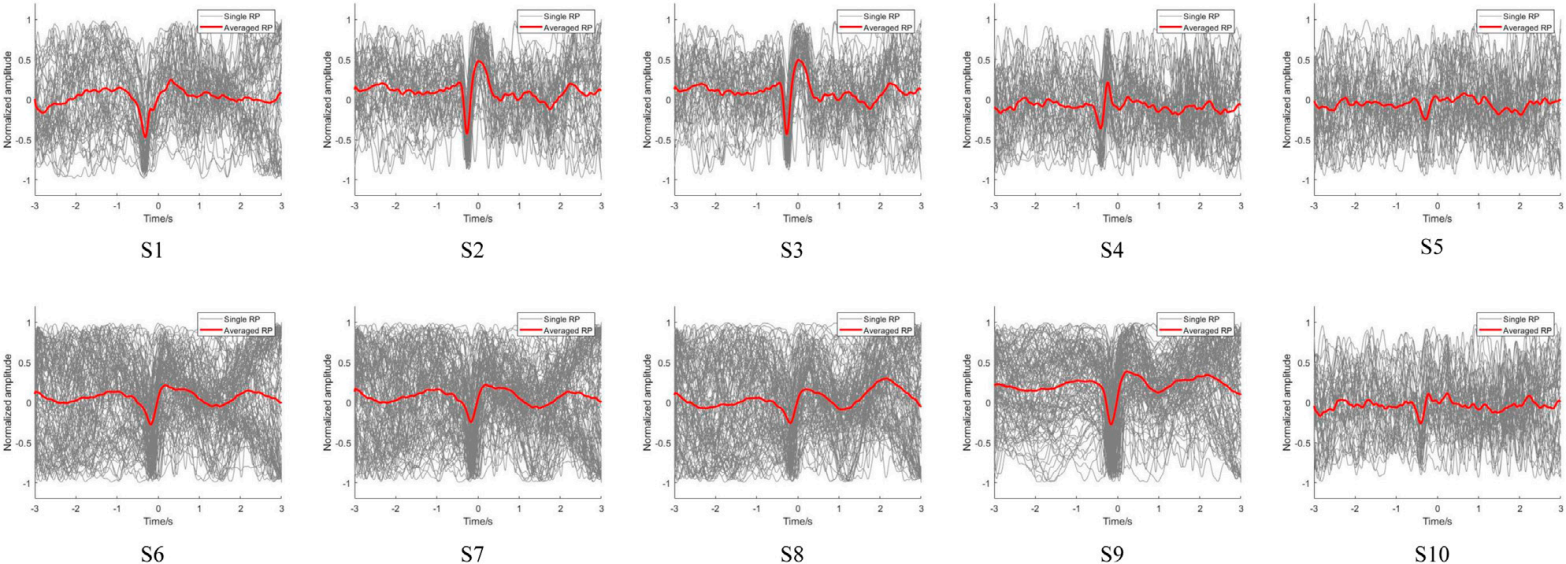
RP results of voluntary right leg movements in the 10 subjects.

Using the above onset trigger detection method, the EMG-based movement onset trigger was set as zero time to distinguish the potential changes in the EEG signals. After preprocessing the acquired signals, the low-frequency EEG signals of the participants were extracted to observe their RP features. The EEG signals of the subjects during voluntary lower limb movements at FC1 are shown in Figure 7. The EEG signals of all subjects showed RP features upon completion of the fall and recovery of the negative potential before onset of voluntary movement, which illustrates the ubiquity of the RP features. However, the response magnitudes varied between the subjects, and we observed that the reactions of S5 and S10 was weaker than those of the other subjects. This may be because the RP is susceptible to various factors, such as preparatory state, level of intention, praxis movements, perceived effort, and individual differences in the brain structure (Shakeel et al., 2015; Berchicci et al., 2020). Moreover, as with most studies, the single RPs of the subjects were not significant, implying that direct feature extraction will not be effective and that deep learning of the features is required.

**FIGURE 8:**
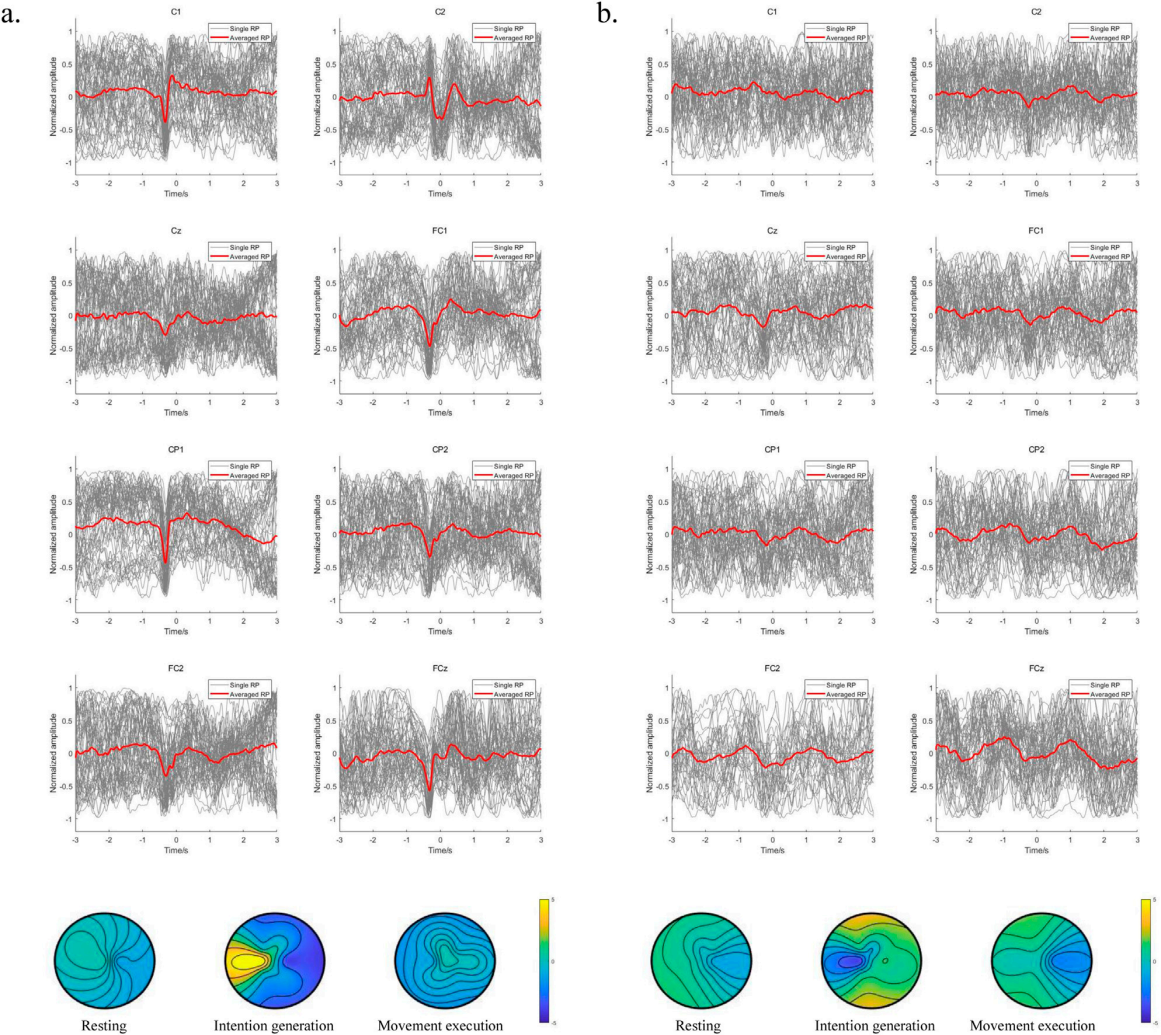
**(A)** Averaged RP from the right leg movements of S1; **(B)** averaged RP from the left leg movements of S1.

To further compare the variability of EEG signals from the motor cortex of the brain between left and right leg movements during voluntary lower limb movements in the subjects, the EEG signals from the central motor areas FCz, FC1, FC2, Cz, C1, C2, CP1, and CP2 were analyzed in detail. We also compared the brain topographies for the resting, intention generation, and movement execution states. The averaged RP is observed for the lower limb voluntary movement from S1 shown in Figure 8. Figure 8A is the result of right leg movements, and Figure 8B is the result of left leg movements; the gray line indicates the single RP, while the red line indicates the averaged RP. It can be seen that the EEG signals from all channels of S1 showed RP features during voluntary movement of the right leg, and most of the channels also showed RP features during movement of the left leg. Moreover, the RP responses of right leg movements were significantly higher than those of left leg movements in terms of the response amplitudes and response times. This may be because all subjects were more accustomed to using their right legs in daily life. In addition, the low-frequency potentials of channels CP2 and Cz during left leg movements were unchanged, which may be attributed to the weak RP features of the left leg movement itself as well as proximity of this channel to the reference electrode channel CPz, resulting in insignificant changes in these channels. Observations from the brain topography showed that there was a significant decrease in the signal amplitude during the intention generation state, with a somewhat larger change in intention for right leg movements than for left leg movements that was accompanied by a weak but insignificant contralateral RP response and no significant changes in the resting and movement execution states. The variability of the EEG signals for left and right leg movements as well as variability between the different channels during movements pose challenges for intention detection and prediction, meaning that a data migration approach may be needed to improve the efficiency when using neural networks.

The results indicate that the motor area is responsible for lower limb voluntary movements; further, RP responses exist in the EEG signals from the motor cortex during the lower limb voluntary movements of the subjects, and these occur before the movements. The long latencies of the RPs allow enough reserve time for feature extraction, pattern recognition, and robot system response, all of which contribute to robot control. The insignificant single RP and individual differences in some subjects are the key points in predicting lower limb voluntary movement intention. Specifically, in the context of exoskeleton robot interaction and control, the experimental results provide a foundation for lower limb movement intention prediction and compliant control of an exoskeleton robot.

### 4.2 Offline results of movement intention detection

**FIGURE 9:**
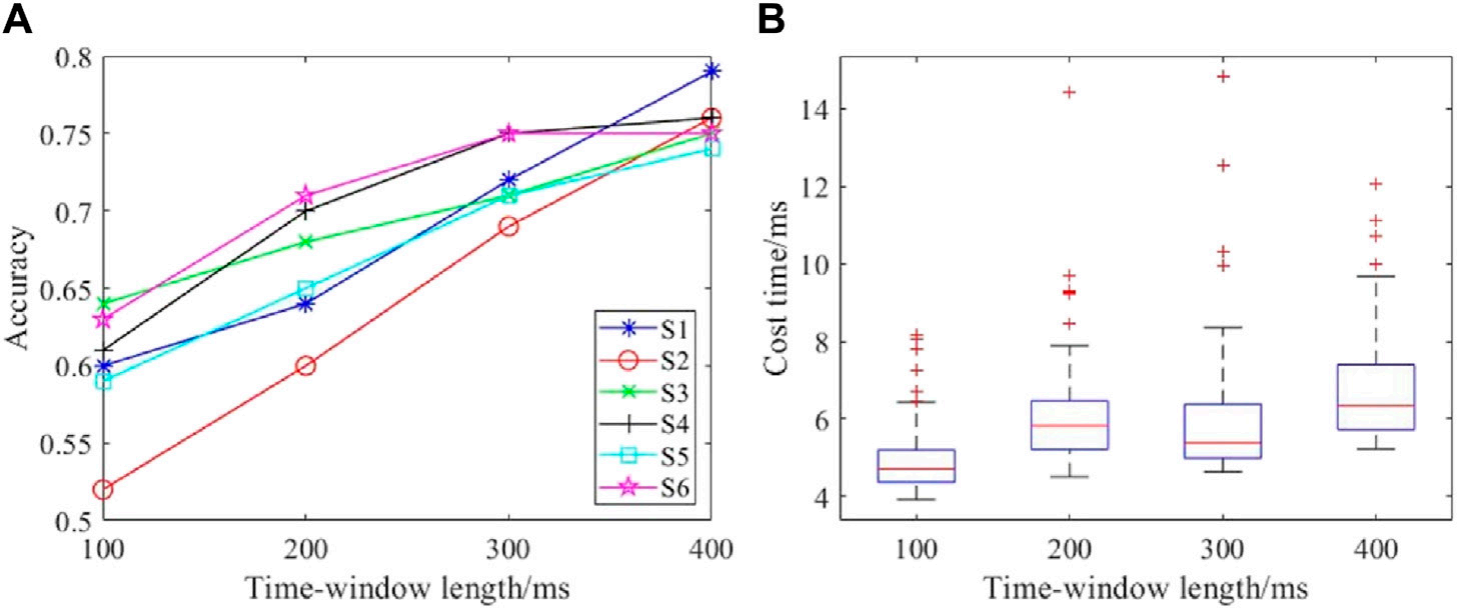
Performances with different time window lengths: **(A)** detection accuracies with different time windows; **(B)** time costs with different time windows.

The signal length greatly influences the detection performance. The comparison results for different data lengths are shown in Figure 9. Figure 9A shows the accuracies for different time windows, and Figure 9B presents the costs for time spent on the computational process. The accuracy increases with increase in the time window length, and this observation was consistent among the subjects. The time cost of single detection was about 6 ms for varying lengths of the time window. Although some single detection tasks required longer times, a two-factor ANOVA showed that there was no significant difference for different time window lengths (*F* = 0.6666, *p* = 0.4152). Therefore, the time window length of the EEG data was set to 400 ms for both accuracy and real-time performance.

Accuracy, recall, precision, and F1 score were used as the metrics to evaluate the performance and were calculated as Equations 9–12.

$$Accurary=\frac{\left(TP+TN\right)}{\left(TP+FN+FP+TN\right)}$$ (9)

$$\text{Re}call=\frac{TP}{\left(TP+FN\right)}$$ (10)

$$Precision=\frac{TP}{\left(TP+FP\right)}$$ (11)

$$F_{1}-score=\frac{2\cdot Recall\cdot precision}{Recall+precision}$$ (12)

where TP is the true positive, FN is the false negative, FP is the false positive, and TN is the true negative.

The test results of the proposed model for the 10 subjects are shown in Table 1. It is seen that the average accuracy ± standard deviation of the right and left leg voluntary movements using the proposed model are 95.23% ± 1.25% and 91.21% ± 1.48%, respectively. Moreover, the recall, precision, and F1 score metrics of the model are balanced, with all three metrics exceeding 90% and 85% for the right and left leg voluntary movement intention detection, respectively, implying that the proposed model performed well.

**TABLE 1:** Detection results and performance comparisons.

Subjects	Right leg				Left leg			
	Accuracy (%)	Recall (%)	Precision (%)	F1 score (%)	Accuracy (%)	Recall (%)	Precision (%)	F1 score (%)
S1	95.00	92.93	92.00	92.46	90.07	83.65	87.00	85.29
S2	96.67	94.12	96.00	95.05	92.00	89.58	86.00	87.76
S3	96.00	93.14	95.00	94.06	91.33	90.22	83.00	86.46
S4	95.67	94.85	82.00	93.40	89.67	82.24	88.00	85.02
S5	93.67	90.10	91.00	90.55	89.33	84.69	83.00	83.84
S6	93.33	90.82	89.00	89.90	90.67	86.00	86.00	86.00
S7	97.67	96.04	97.00	96.52	93.00	89.11	90.00	89.55
S8	94.67	93.75	90.00	91.84	94.33	91.92	91.00	91.46
S9	94.67	91.18	93.00	92.08	90.33	83.81	88.00	85.85
S10	95.00	91.26	94.00	92.61	91.33	83.64	92.00	87.62
Mean ± Std	95.23 ± 1.25	92.82 ± 1.84	92.90 ± 2.47	92.85 ± 1.89	91.21 ± 1.48	86.49 ± 3.24	87.40 ± 2.91	86.89 ± 2.16

**FIGURE 10:**
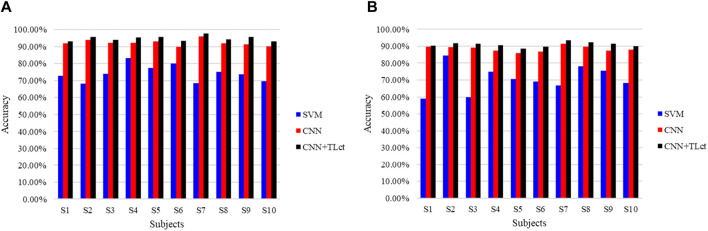
Comparison of the cross-temporal detection accuracies of different methods for **(A)** right and **(B)** left leg movement intentions.

To further validate the benefits of TL in the proposed model, we asked the subjects to do the same walking experiment again after a month’s interval and used the new data as the inputs to the already trained network to obtain the detection accuracy, as shown in Figure 10. The traditional SVM, CNN, and proposed combination of CNN and TL methods are compared. Figure 10A shows the right leg movement intention detection accuracy, and Figure 10B shows the left leg movement intention detection accuracy. In the figure, the results using the SVM approach are shown in blue, results using only the CNN without TL are shown in red, and results using the proposed method are shown in black; the TL for the cross-temporal case is denoted by TL_ct_ here to distinguish it from cross-subject TL. It is seen that the accuracy of cross-temporal voluntary movement intention detection using SVM is the lowest, with only 74.3% ± 4.70% for the detection of right leg movements and 70.68% ± 7.48% for left leg movements. The detection accuracy using CNN was substantially better, reaching an average accuracy of 92.33% ± 16.5% for the detection of right leg movements and 88.45% ± 1.57% for the detection of left leg movements. These results indicate that features that are not obvious in a single EEG can be learned better using deep-learning methods. The accuracies of all the participants improved when using CNN combined with TL, with the detection of right leg movements eventually reaching an average accuracy of 94.77% ± 1.42% and that of left leg movements reaching an average accuracy of 90.93% ± 1.29%. In addition, the training losses of all subjects decreased after applying TL, indicating that TL can effectively improve the performance of cross-temporal movement intention detection.

**FIGURE 11:**
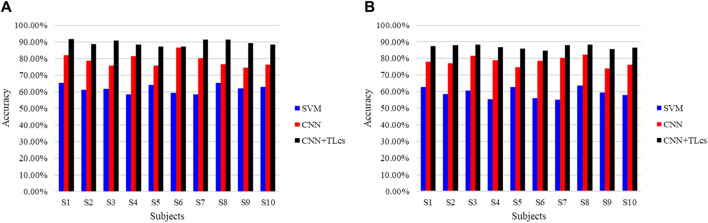
Comparison of the cross-subject detection accuracies of different methods for **(A)** right and **(B)** left leg movement intentions.

We also evaluated the proposed model for cross-subject performance. Each subject was tested using a network trained on data from the other subjects, and the results are shown in Figure 11. As with the cross-temporal test, we compared three different approaches. Figure 11A shows the right leg movement intention detection accuracy, and Figure 11B shows the left leg movement intention detection accuracy. In the figure, to distinguish the TL approach for the cross-temporal case, TL_cs_ was used to denote cross-subject TL. The results show that for cross-subject voluntary movement intention detection, the accuracy of detection of right leg movements using the SVM method was 61.83 + 2.49% and that of left leg movements was 59.20% ± 3.01%. The right leg movement evaluation detection accuracy using the CNN method was 78.80% ± 3.58%, while that of the left leg movements was 78.20% ± 2.64%. The results of these two methods are unsatisfactory, indicating that the learned features are highly specific and weakly generalized for both the machine-learning and deep-learning processes. Moreover, the accuracy obtained when using the proposed CNN combined with TL is much higher than those when using the SVM and CNN methods. The average accuracy of right leg motion detection reached 89.43% ± 1.60%, and the average accuracy of left leg motion detection reached 87.01% ± 1.19%. This implies that TL offers considerable advantages across subjects.

The elapsed training time was also calculated when the target domain data were used as inputs to the network. The average elapsed training times and their standard deviations were 1,057 ± 185 s for the CNN and 745 ± 164 s for the proposed methods. Thus, the proposed method required about one-third less training time than the CNN method; hence, TL greatly improved the efficiency of training while ensuring high accuracy. In summary, the results show that the proposed CNN combined with TL can enhance the network performance, obtain high accuracy, show good generalization for both cross-temporal and cross-subject aspects, and reduce the training time without compromising on accuracy.

### 4.3 Online performance of movement intention prediction

**FIGURE 12:**
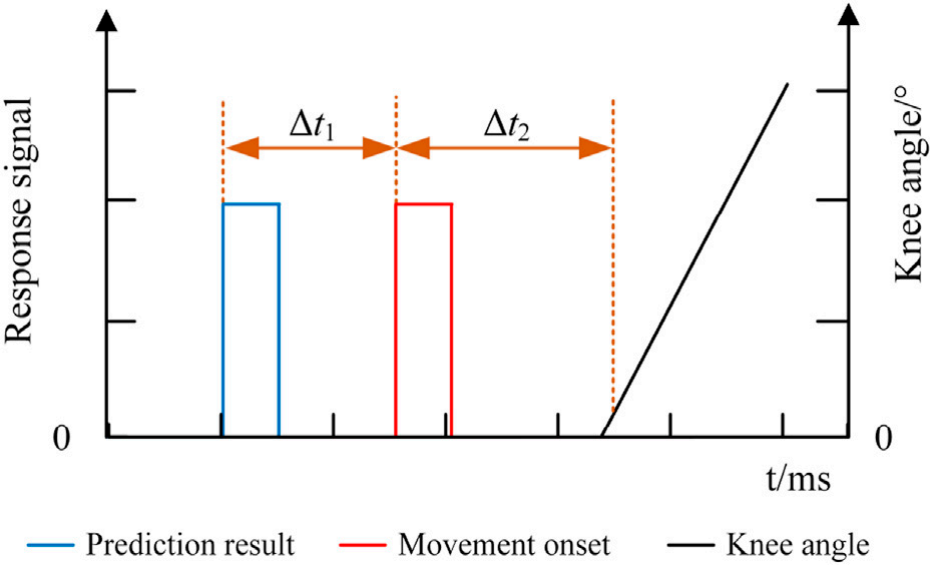
Response times of the prediction system.

The responses of the EEG, EMG, and knee angle signals of the exoskeleton robot were recorded in the online experiment, and the response time of each signal is shown in Figure 12. The blue line indicates the predicted result of lower limb voluntary movement intention based on the EEG signal, the red line indicates the onset of lower limb voluntary movement based on the EMG signal, and the black line indicates the knee angle signal of the lower limb exoskeleton robot. The response time differences between the signals were calculated using the movement onset as the reference. Δ*t*_1_ indicates the time difference between the predicted result and movement onset, while Δ*t*_2_ indicates the time difference between the knee angle of the robot and movement onset. In this experiment, Δ*t*_2_ was the time required by the robot system for information processing and response since the subjects were not wearing the exoskeleton robot. The response time was determined by performing an unloaded test on the exoskeleton robot. The results were averaged over five repeated trials to minimize the effects of random errors, and the results showed that Δ*t*_2_ was approximately 40 ms. The movement onset marked by the EMG signal was noted as moment 0. The prediction time should be ahead of the motion onset moment; therefore, in the experiments, instances whose prediction times were less than or equal to zero (Δ*t*_1_≥0) were marked as wrong predictions, and their prediction times were set to zero. The prediction accuracies and prediction times were statistically analyzed for all subjects.

**FIGURE 13:**
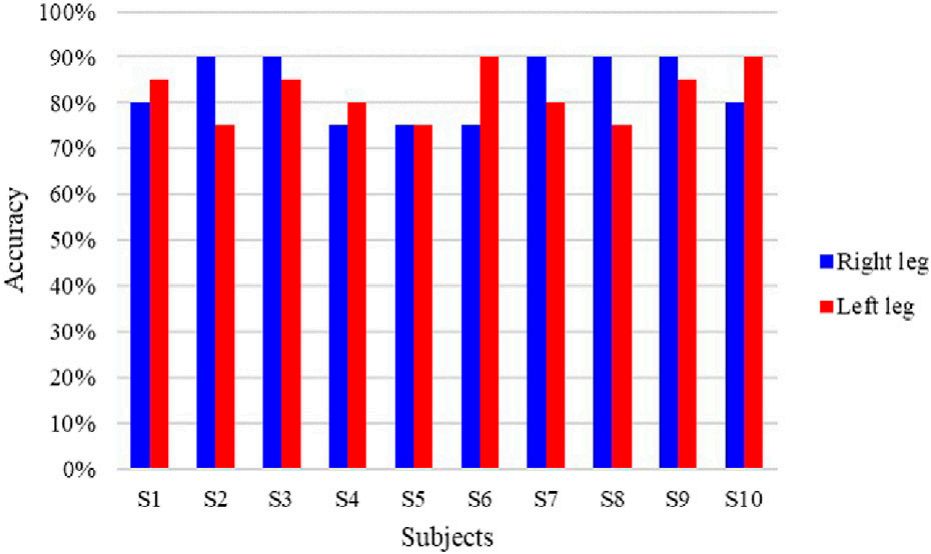
Prediction accuracies of the online experiment.

**FIGURE 14:**
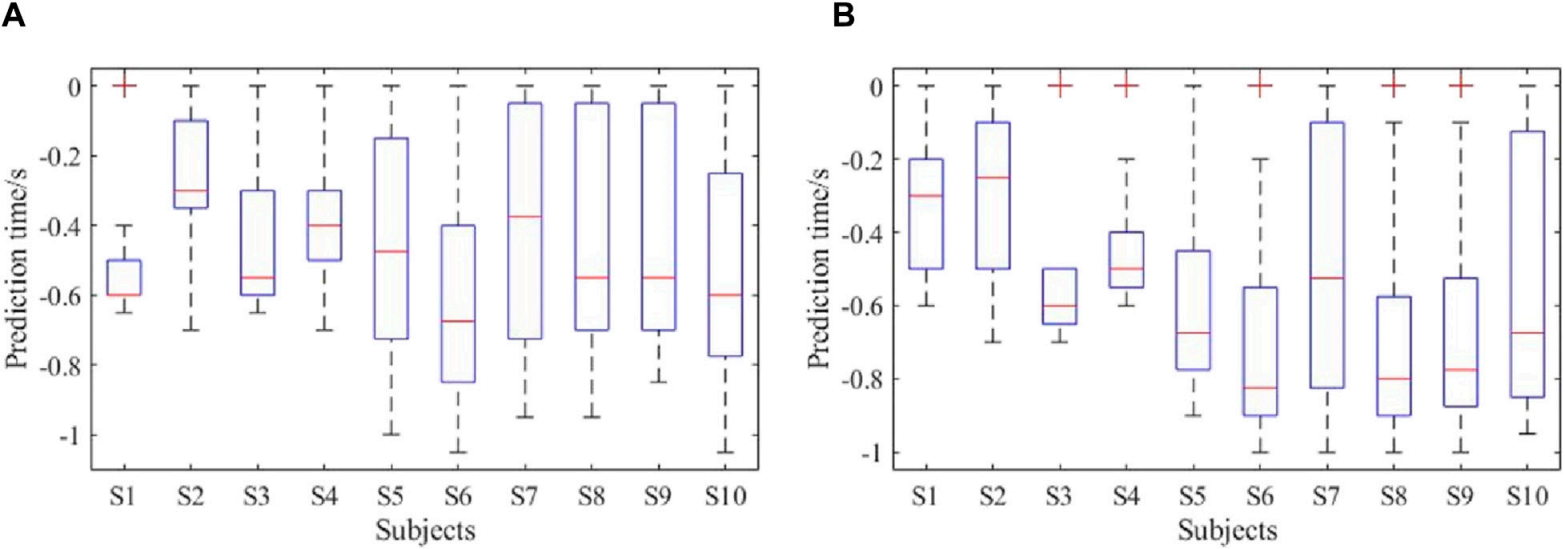
Prediction times of the online experiment: **(A)** performance of right leg movement; **(B)** performance of left leg movement.

The prediction accuracies of all the subjects are shown in Figure 13. As seen, the average lower limb voluntary movement intention prediction accuracy of the left leg was 82% ± 5.6%, and the average prediction accuracy of the right leg was 83.5% ± 6.7%. The highest accuracy rate was 90%, while the lowest was over 75%. Although there were some variations between the subjects, a two-tailed *t*-test showed no significant difference in the prediction accuracies between the lower limb voluntary movements (*t* = 0.461, *p* = 0.66). The statistical results of the prediction time are shown in Figure 14, where the Δ*t*_1_ can be seen to vary from −710 to −272.5 ms with a median value of −470 ms and average prediction time of −483.9 ± 11.9 ms. This result indicates that the proposed method can effectively predict the intention of lower limb voluntary movement. Further, the prediction time exceeds 40 ms for information processing by the exoskeleton robot, which provides a good foundation for interactive control of the exoskeleton robot. Although the results of a two-factor ANOVA showed significant differences in the prediction times between different subjects and lower limb voluntary movements (*F* = 3.8959, *p* = 0.0092), all results were adequate for the exoskeleton robot control response.

Based on the online experimental results, the proposed method realized good prediction of lower limb voluntary movement intention, and its prediction accuracy and prediction time were satisfactory, which provide a good foundation for exoskeleton robot control. To further highlight the advantages of the proposed method, the statistical results of the prediction study of movement intentions of the relevant lower limbs are shown in Table 2. These results indicate that the prediction of limb movements using EMG or angle signals has a short latency, which poses challenges to the robot’s responses. Using the feature RPs of the EEG signals to predict limb movement intentions required longer prediction times but tended to be less accurate. Our approach was able to achieve high online accuracy at longer prediction times. Overall, the method proposed in this study shows excellent performances for both offline and online experiments and is able to maintain highly accurate outputs before movement execution, laying the foundation for further investigations of the control responses of exoskeleton robots or related peripherals.

**TABLE 2:** Comparison of studies related to the prediction of movement intentions.

Reference	Target	Feature	Classifier	Accuracy	Prediction latency (ms)
Lew et al. (2012)	Upper limb	RP	LDA	76%	−167
Buerkle et al. (2021)	Upper limb	RP	LSTM-RNN	84.98%–92.08%	−513 to −54
Wöhrle et al. (2017)	Upper limb	RP + EMG	SVM	75%	−269
Feleke et al. (2021)	Upper limb	EMG	RFNN	Pearson cc = 0.85	≥ −250
Tortora et al. (2019)	Upper limb	EMG	Gaussian mixture model	94.3%	−160 to −80
Faquan and Jun (2020)	Lower limb	Angle	Multidimensional temporal association	78.3%	−92.24
Hasan et al. (2021)	Lower limb	RP	SVM	9/12 (75%)	−741
Our study	Lower limb	RP	CNN-TL	Offline: 93.22% Online: 82.75%	−710 to −272.5

## 5 Discussion

### 5.1 Movement intention prediction performance

Based on the multiple-input and multiple-output model of brain cognition, the EEG and EMG signals are homologous in their generation but differ in their signaling and response times. Therefore, when using the EEG RP features to predict lower limb voluntary movement intentions, it is scientific to use the surface EMG signals that characterize the activation of lower limb skeletal muscles as the basis for judging the onset times of lower limb voluntary movements; this is because the true moment of onset of human voluntary movement is the only method of evaluating the predictive ability of intention.

The detection results of RPs show that they have long latencies and can provide a good basis for prediction of lower limb voluntary movement intentions. By observing the EEG RP features of the 10 subjects, it is noted that the single RPs produced during voluntary lower limb movements of the human body are not significant but the multitrial superposition effect is significant, which is consistent with the findings of other studies in literature (Seneviratne and Gilwon, 2020). Based on observation of the brain topography with multichannel EEG data, we found that there was a contralateral RP response to lower limb movements that was not obvious; however, this trend was consistent across all channels. This finding is consistent with a reported study on EEG signals from different movement sites on the human body, which compared the RP features of tongue, hand, leg, and ankle movements; it was observed that the farther the body site was from the brain, the higher was the contralateral RP response of the movement toward the midline. Moreover, we observed that the RP features generated by the right leg voluntary movements were significantly more pronounced than those of the left leg movements, which may be related to the fact that the dominant leg of all the subjects was the right leg during the free movements. However, the reason for its neural connectivity needs to be investigated further.

The offline detection accuracy demonstrated the efficiency of the proposed method. Many studies have shown that the recognition accuracies of lower limb movements are lower than those of the upper limbs and that the recognition accuracies of voluntary movements are lower than those of evoked movements when decoding motor intention based on EEG signals. The method proposed in this study achieves more than 90% accuracy for lower limb voluntary movement intention prediction and performs well in terms of the recall, precision, and F1 score metrics while outperforming traditional machine-learning and single deep-learning methods. Although the insignificant characteristics of single-trial RPs and individual differences in the EEG signals show differences in the detected results between subjects, the experimental results exceed the performances of existing studies and prove the validity of the proposed method. Moreover, the proposed method shows excellent cross-temporal and cross-subject performances, reducing the network training time considerably while maintaining high accuracy; this is expected to offer a foundation for the practical applications of BCI intention prediction techniques.

Studies have shown that a single-trial detection accuracy of 70% or more is required for all subjects to use the BCI system to control the exoskeleton robot. In the online experiments, the lowest accuracy was 75% and average accuracy was 82.75%, which is in line with this consensus. The movement intentions can be predicted up to 272.5–710 ms before motion onset with the proposed method. This is a significant time lead that cannot be achieved with other signals, such as EMG or angle signals, with equal prediction accuracy. Moreover, the proposed method provides a higher accuracy than other methods that offer the same lead on prediction time. This prediction time lead offers enough reserve time for information processing by the exoskeleton robot, which can help realize interactive control to overcome the problem of response time lag for the user. In summary, the RP-based prediction method for lower limb voluntary movement intention proposed in this study has good prediction accuracy and is well-prioritized in terms of the temporal responses, thereby laying the foundation for further studies on exoskeleton robot control.

### 5.2 Limitations of the study and further work

The main limitation of this study is that the subjects did not wear the exoskeleton robots to validate the online prediction performance. When subjects wear the exoskeleton robot, more noise will be present in the EEG signals, which may increase the difficulty of RP detection and movement intention prediction via EEG signals. The experiments did not involve an asynchronous control method for real testing. Hence, future works will involve combining the exoskeleton control with asynchronous BCI. There is no doubt that the main motivation for this work was to provide a novel RP-based BCI system for controlling an exoskeleton robot to solve the real-time performance between human movement intention and robot response. The results presented in this work are based on the feasibility of RP-BCI usage in robot control. Future studies will also involve more subjects using the RP-BCI system to control a robot.

Another limitation of this study is the gender imbalance among the subjects, even though no differences were found between the male and female subjects in the experimental results. Thus, a large sample size and balance between the numbers of male and female subjects are desired to fully evaluate the robustness of the proposed method and system. Furthermore, as stroke survivors are the potential targets of this study in terms of people requiring appropriate robot control, we expected more new challenges when predicting their active movement intentions because of differences in their types and areas of brain injuries; this gap should be further addressed in future studies. In future work, another BCI paradigm will be added to build a hybrid BCI system for application. The proposed voluntary movement detection scheme without external stimulation is also expected to play an important role in lower limb neurorehabilitation, which can help improve the autonomy of patients in the process of rehabilitation; however, the EMG signal specificity of some patients still needs further research.

## 6 Conclusion

Targeted control of exoskeleton robots requires early prediction of human lower limb movement intentions to handle any constraints caused by control delays. Based on the principles of human intention generation and movement expression processes, EEG signals were used to predict human lower limb voluntary movement intention in this study. Based on the susceptible and single-trial non-obvious properties of EEG RP features as well as cross-domain problems of BCIs in practical applications, a VGG-based CNN framework combined with TL-based prediction was proposed for lower limb voluntary movement intention. Surface EMG signals were used to mark the voluntary movement onset moment, and the MEMD method was used to remove artifacts. The proposed method quickly learns the pretrained model and transfers the parameters to a new target domain, thus effectively solving the cross-temporal and cross-subject problems involved in applying BCI intention prediction online. Offline and online experiments were conducted to validate the good performance of the proposed method. Overall, the proposed method required less training time and produced high prediction accuracy and good generalization, along with a larger prediction time lead for exoskeleton robot responses. This is a significant criterion for interaction control of lower limb exoskeleton robots.

## Data Availability

The original contributions presented in the study are included in the article/Supplementary Material, further inquiries can be directed to the corresponding author.
